# Mussel Culture Farming Systems in the Northern Gargano Coast (Adriatic Sea): Changes in the Nutritional Profile of the *Mytilus galloprovincialis*

**DOI:** 10.3390/foods13142205

**Published:** 2024-07-13

**Authors:** Antonella della Malva, Antonella Santillo, Matteo Francavilla, Mariangela Caroprese, Rosaria Marino, Agostino Sevi, Marzia Albenzio

**Affiliations:** 1 Department of Agriculture, Food, Natural Resources and Engineering (DAFNE), University of Foggia, 71121 Foggia, Italy; antonella.santillo@unifg.it (A.S.); matteo.francavilla@unifg.it (M.F.); mariangela.caroprese@unifg.it (M.C.); rosaria.marino@unifg.it (R.M.); agostino.sevi@unifg.it (A.S.); marzia.albenzio@unifg.it (M.A.); 2 STAR Integrated Research Unit, University of Foggia, 71121 Foggia, Italy

**Keywords:** mediterranean mussels, farming methods, aquaculture, Gargano area, fatty acids, amino acids

## Abstract

The objective of this study was to investigate the nutritional characteristics of *Mytilus galloprovincialis* cultivated in three sites of the Northern Coastal Area of Gargano. The mussel farms were characterized by different distances of dropper ropes (extensive dropper ropes: EDR; intensive dropper ropes: IDR; semi-intensive dropper ropes: SIDR). Mussels’ chemical composition, fatty acids, and amino acids profiles were investigated at monthly intervals, from April to June. The fat content of mussels from EDR and SIDR sites was lower than values observed for mussels of the IDR in both the April and June sampling months. An increase in the main saturated fatty acids (C15:0; C16:0; C17:0 and C18:0; *p* < 0.001) and polyunsaturated fatty acids (C18:2c9c12, C18:3n3, C20:2n6, C20:4n6, and C22:6n3; *p* < 0.001) was found in the mussels collected in June from all farms analyzed. In terms of farming systems, mussels from the EDR and SIDR sites exhibited the greatest content of beneficial molecules like conjugated linoleic acids isomers (*p* < 0.001), glutamic acid (*p* < 0.05), serine (*p* < 0.05), tryptophan (*p* < 0.001), hydroxyproline (*p* < 0.05) and proline (*p* < 0.01). This study revealed that the farming system can affect the nutritional composition of mussels and evidenced that EDR and SIDR are the most effective cultured farming methods in the Gargano area.

## 1. Introduction

Marine aquaculture is considered a promising sector in the food industry around the world, with growth projections from 66.6 million tons in 2012 to 93.2 million tons by 2030 [1]. Of total global marine food production, mussel farming accounts for 19% in the Mediterranean region [2]. Currently, mussel farms in the Gargano areas are a thriving industry, a result of a tradition of ancient origin, contributing to the region’s economy as well as providing a sustainable source of high-quality products. Indeed, the Gargano Peninsula, located in the Apulia region of Southern Italy, offers an ideal environment for mussel cultivation due to its favorable climate, nutrient-rich waters, and extensive coastline. Mussel farms have a minimal ecological impact because bivalves can obtain feed, autonomously, via the environment (i.e., filter feed), whereas fish farms can accumulate high amounts of organic matter as a consequence of feed supplements [3].

Offshore mussel farms represent a strategic and effective bio-based system that provides efficient recycling of nutrients from sea to land by eliminating nutrients from eutrophic waterways [4]. In the perspective of achieving the 2030 Agenda goals for sustainable development, finding appropriate farming strategies/techniques and areas for the expansion of sustainable marine aquaculture is essential to protect the coastal–marine ecosystem as well as to improve the aquaculture sector. Intensive mussel culture systems have been adopted in many coastal areas of Southern Europe [5], having a significant impact on environmental conditions and the ecosystem due to changes in water salinity and temperature, and rearing density [6]. Longline systems are considered to be the most practical method for expanding bivalve aquaculture into offshore locations [7]. A longline system consists of a horizontal main line (backbone) with numerous dropper lines hanging from it. Buoyancy is provided to the backbone and dropper lines by using buoys or other flotation devices. The entire system is typically anchored to the sea bottom. The density of mussels in farm systems is a critical factor influencing their survival, growth, and overall productivity. The range of densities employed in mussel aquaculture can vary significantly based on numerous factors, including the specific cultivation method, the environmental conditions, and the objectives of the aquaculture operation. Extensive mussel farming generally has a relatively low impact on coastal–marine ecosystems due to farming practices based on the natural food/water resources and surrounding ecosystem to support their growth, with minimal human intervention [8]. However, although extensive mussel systems have several benefits, taking into account the site-specific conditions and challenges such as variable growth rates and vulnerability to environmental fluctuations may be crucial to ensuring the sustainability of these farming practices.

Therefore, considering recent global environmental concerns and animal welfare issues, exploring and defining the nutritional quality differences between aquaculture products originating from different farming sites should be fundamental to better understanding the biological processes and the chemical–physical conditions characterizing farming sites that can lead to mussels’ quality variations. However, to the best of our knowledge, at present no studies have investigated the effect of farming culture conditions and, particularly, the mussels’ stocking density, on their nutritional value with the objective of defining more appropriate strategies and practices linked with a specific geographical area. Mussels’ aquaculture provides food products with high-quality proteins that represent an important part of the Mediterranean diet, especially in health-conscious consumers [9]. In this regard, exploring and optimizing mussels from different farming systems in the Gargano area, characterized by different rearing conditions and environmental impact, should be of interest to both farmers and consumers to find and propose strategies for improving local mussels’ sustainable production as well as to improve the ecosystem and biodiversity.

Therefore, the aim of this study was to assess the nutritional characteristics of *Mytilus galloprovincialis* harvested in the Northern Coastal Area of Gargano, Southern Italy, in three different sites characterized by different farming methods.

## 2. Materials and Methods

### 2.1. Study Area and Sampling Sites

Mediterranean mussels, *Mytilus galloprovincialis*, were collected from three different farming sites, Site A, Site B, and Site C, located about 2.1 km along the Northern Coastal Area of Gargano (Apulia region, Southern Italy, Central Mediterranean Sea). The Gargano area is characterized by several economic uses and activities carried out even in natural contexts, given by natural national and regional marine parks. The mussel farms were selected based on the most important aquaculture facilities as well as to monitor and analyze mussels from farming methods representative of those cultivated by mussel farmers along the Gargano Coastal Area.

**Figure 1:**
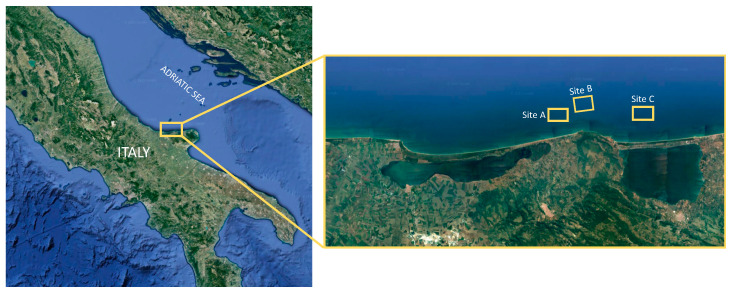
Map of the Adriatic Sea with zoomed-in section of the Gargano coast representing the location of the studied farming sites (Site A: EDR; Site B: SIDR; Site C: IDR. Distance between sites: Site A–Site B: 3.5 km; Site B–Site C: 7.3 km; Site A–Site C: 10.3 km).

Geographical areas and sampling sites from which mussels were collected are indicated in Figure 1, as follows:

- Site A (N 41°56.4359′; E 015°34.7990′) was characterized by an extensive mussel dropper ropes farming method (EDR; 70 m distance between backbones; 90 cm distance between dropper ropes);
- Site B (N 41°56.8906′; E 015°37.1863′) was characterized by a semi-intensive mussel dropper ropes farming method (SIDR; 70 m distance between backbones; 50/60 cm distance between dropper ropes);
- Site C (N 41°56.3240′; E 015°42.5785′) was characterized by intensive mussel dropper ropes farming (IDR; 30 m distance between backbones, 60/70 cm distance between dropper ropes).

In each site, seeding was carried out in September 2022 with wild seeds collected from ropes, resulting in similar sizes and densities. Mussels were sampled in the three farming sites from April to June 2023, during the spring season.

At each sampling, 2 kg of mussels (40 animals per site) was collected at 10 m depth from each sampling site (every platform). After collection, mussels were immediately transported to the laboratory of the University of Foggia in polyethylene bags with seawater. Once in the laboratory, mussels were rinsed with Milli-Q water and then the byssus and shells were removed. Soft tissues were stored in vacuum-sealed bags at −20 °C until the subsequent analysis. Before each analysis, soft tissues were homogenized (pools of 10 animals) by means of an Ultra-Turrax disperser (IKA T25 easy clean control, Staufen, Germany).

### 2.2. Chemical and Physical Seawater Analysis

Chemical and physical characteristics of seawater were monitored across three chosen locations (farming sites) during the sampling period to provide information on the global water quality. Temperature (°C), salinity (psu), dissolved oxygen (DO, % saturation) and pH were measured, directly in the field, by using multi-parameter instruments (Aquaprobe AP-5000 and GPS Aquameter multiprobe system, supplied by AQUAREAD Ltd., Broadstairs, UK). Data are expressed as mean values gathered on both, at the surface and throughout the water column.

### 2.3. Chemical Composition and Fatty Acid Methyl Esters (FAME) Profile

Moisture, protein, lipid and ash contents were analyzed according to AOAC methods [10]. All the chemical determinations were performed in triplicate.

Analysis of fatty acid composition was conducted following O’Fallon et al.’s [11] protocol, with some modifications. Briefly, 1 g of each sample was placed into a screw cap Pyrex reaction tube, with 5.3 mL of MeOH, 0.7 mL of 10 N KOH in water, and 0.5 mg of C13:0/mL of the internal standard added. Then, the tubes were incubated in a water bath at 55 °C for 90 min, with hand shaking for 5 s every 20 min. After incubation, the tubes were cooled at room temperature, and subsequently, 580 μL of 24 N H_2_SO_4_ was added. The tubes were mixed by inversion and incubated again, as previously described. After cooling, 3 mL of hexane was added into each tube, vortexed on a multitube vortex for 5 min, and then centrifuged at 500× *g* (Eppendorf 5810 R, Eppendorf AG, Hamburg, Germany) for 5 min at 21 °C. The hexane layer containing the fatty acids methyl esters (FAME) was collected and transferred into a gas chromatographic vial. The fatty acids’ profile was quantified through an Agilent 6890 N instrument (Agilent Technologies, Santa Clara, CA, USA) equipped with an HP-88 fused-silica capillary column (length 100 m, internal diameter 0.25 mm, film thickness 0.25 μm). The carrier gas was helium with a constant flow rate of 1 mL min^−1^ and a split ratio of 1:25. The injector temperature and FID detector were set at 260 °C. The temperature program of the column oven was initially programmed at 100 °C for 5 min, then increased to 240 °C (3.5 °C min^−1^) and held/maintained for 15 min. The retention time and area of each peak were computed using the Chemstation software B04.03 (Agilent Technologies, Santa Clara, CA, USA). Fatty acids were identified by comparing their retention times with the fatty acid methyl standards (FIM-FAME-7-Mix, Matreya LLC, State College, PA, USA), with C18:1,11t, C18:2 9c,11t, and C18:2 9t,11t (Matreya LLC, State College, PA, USA) added. The fatty acid concentration was expressed as g fatty acids/100 g total fatty acids.

### 2.4. Amino Acid Composition

The amino acid composition of mussels was determined according to the procedure described by Marino et al. [12]. Briefly, 100 mg of each mussel sample was placed into a screw cap Pyrex tube with 500 μL of 6 M HCl and hydrolyzed at 160 °C for 75 min. After acid hydrolysis, samples were filtered through Whatman 0.45 μm filters and subsequently diluted 1:10 with ultrapure water before being submitted to automated online derivatization and injection. Identification of amino acids was carried out by using an Agilent 1260 Infinity Series chromatograph HPLC system (Agilent Technologies, Santa Clara, CA, USA), equipped with a reversed-phase Zorbax Eclipse AAA column (150 × 4.6 mm i.d., 3.5 μm particles; Agilent Technologies, Palo Alto, CA, USA), a binary pump (G1312B), a diode-array (1315C), and fluorescence (G1321B) detectors. Individual amino acids peaks were identified by comparing their retention times with those of standards. The amino acids content was expressed as mg/100 g meat.

### 2.5. Statistical Analysis

All data were subjected to analysis of variance (ANOVA) using the GLM procedure of the SAS statistical software, version 6.1 [13]. The mathematical model included the fixed effects of farming system, sampling time, farming system × sampling time, and random residual error. All effects were tested for statistical significance set at *p* < 0.05, and when significant effects were observed (*p* < 0.05), Tukey’s test was used for post hoc comparison.

## 3. Results and Discussion

### 3.1. Chemical and Physical Seawater Parameters of the Monitored Sites in the Northern Coastal Area of Gargano

The environmental parameters of seawater from each of the three farming sites of the Northern Coastal Area of Gargano recorded during the spring season are presented in Table 1. Among these, only pH did not show any notable differences, whereas the water temperature, salinity, and dissolved oxygen (DO) values exhibited notable variations over the sampling period. An increase in the seawater temperature and dissolved oxygen was detected in all the farming sites analyzed from April (15.12 °C and 109.7 for T and DO, respectively) to June (24.70 °C and 120.5 for T and DO, respectively). Conversely, a decrease in the water salinity content was detected in all the farming sites analyzed, varying from a maximum of 30.80 g/kg ppt detected in March to a minimum detected in June of 25.73 g/kg ppt.

### 3.2. Mussels Proximate Composition

Marine aquaculture is a key human activity in Mediterranean coastal areas, as mussels are a fundamental source of proteins for human consumption contributing to the region’s economy. Being filter-feeding organisms, mussels exhibit dynamic and cyclic changes in the concentration of nutritional compounds, especially due to geographical location, reproductive cycle, seasonal changes, climatic variations, and seawater composition [14,15], even when cultured nearby in the same coastal ecosystem or in sites within the same embayment. Therefore, quantifying mussels’ nutritional differences can provide valuable insights into their ecological role and enable informed decisions regarding their conservation and utilization.

Overall, in this study, mussel specimens collected from the Northern Coastal Area of Gargano (Table 2) were characterized by 1.24–2.62% fat, 8.13–10.77% protein, 1.92–3.41% ash, 76.59–81.44% moisture, and 5.14–8.61% carbohydrates, falling within ranges comparable to those reported for Mediterranean *Mytilus galloprovincialis* from different geographical areas [15,16,17]. However, our findings revealed significant differences in the chemical composition of mussels harvested in three sites of the Northern Coastal Area of Gargano during the spring season. In terms of fat content, effects of both the farming system (*p* < 0.01) and sampling time (*p* < 0.001) were found. Considering the mussel farming site, significative differences were found, in the fat content, between mussels collected from EDR and IDR farms in April and June, whereas no significant differences were observed among mussels from the three farming sites in May. It is well known that lipid reserves in mussels are strongly affected by food availability, stocking density, and hydrodynamic patterns [14,18,19]. However, the lower fat content of mussels originating from farms with ropes cultured at lower densities highlights their better nutritional values and confirms that stocking density can affect nutrient dynamics.

Due to sampling time, a significant decrease (*p* < 0.001) in the fat content was observed in mussels collected in June from all farms investigated, evidencing that stressful and unfavorable conditions due to the increase in seawater temperature and the reproductive cycle of the mussels’ may have affected their nutritional profile irrespective of the farming system. However, it is important to underline that, in June, mussels from the EDR farming method showed the lowest fat percentage with respect to the IDR site (1.244% vs. 1.846% in extensive and intensive dropper ropes systems, respectively). In general, lipids constitute the energy reserve in mollusks, and their accumulation or stored reserves can be altered by different factors, including the reproductive cycle, food supply, and seawater temperature [15,17,20]. In particular, during the growth phase (autumn–winter), mussels tend to accumulate lipids, while during the maturation phase/gametogenesis (spring–summer), especially leading up to and during spawning, the lipid stores are mobilized [21]. This mobilization is necessary to support the energy-intensive processes associated with reproduction, such as gamete production and spawning activities. In our study, the lower fat content found in mussels collected from the EDR site of the Gargano area in June could be attributed to both the dynamic energy requirements of mollusks throughout their life cycle and to the variation in seawater temperature (see Table 1).

In our study, no significant differences were detected as a consequence of the farming systems in the protein and ash contents, while an effect of sampling time was found in the content of both nutritional parameters (*p* < 0.001) with an opposite trend. Particularly, an increase in the protein content (*p* < 0.001) was found in all sites reaching higher values starting from May, whereas a gradual decrease in the ash content (*p* < 0.001) was observed. Our results are in accordance with those obtained by Vernocchi et al. [22], who observed remarkable fluctuation and the same trend in the protein and ash contents of Mediterranean mussels from the northern part of Italy due to an increase in seawater temperature.

An effect of both farming system (*p* < 0.001) and sampling time (*p* < 0.001) was detected in the moisture content of mussels collected in the Gargano area, whereas the carbohydrate content was affected only by sampling time (*p* <0.001). Considering the sampling time, a decrease in the moisture content was detected in mussels collected from all farms in May and June; conversely, an increase in the carbohydrate content was found. Generally, carbohydrates represent, together with lipids, an important energy source for maintaining gamete generation and survival during nutritive stress periods (e.g., winter) or the reproductive cycle. In our study, the greater carbohydrate content found in May and June, together with the lower moisture and fat contents detected, could be due to the gametogenesis of the mussels, as previously discussed. Effectively, during the early stage of gametogenesis, carbohydrates are the primary energy source to support gonad development because they can be readily broken down into glucose, which is then used by cells to produce ATP, while lipids may be present in smaller amounts [23,24]. Accordingly, Nikolic et al. [25] reported a prolonged reproductive phase in *Mytilus galloprovincialis* harvested in Boka Kotorska Bay (Montenegro, Adriatic Sea), from October to June, with a brief resting period when the seawater temperature exceeded 25 °C.

### 3.3. Fatty Acid Profile

The effect of the farming system and sampling time on the fatty acid profile of *Mytilus galloprovincialis* harvested in the Northern Coastal Area of Gargano is reported in Table 3. Data revealed a strong influence of farming system and sampling time on the fatty acid profile of mussels collected in the Gargano area. In particular, the sampling time affected the content of the saturated fatty acid (SFA) detected (C14:0; C15:0; C16:0; C17:0; C18:0; Total SFA; *p* < 0.001) but with different trends. In particular, among SFA, only myristic acid (C14:0) showed a decrease due to sampling time, revealing a lower content in June. Our findings are in agreement with a previous study [26] in which a decrease in the content of myristic acid in Tunisian *Mytilus galloprovincialis* was observed due to sampling time and increases in seawater temperature. Taking into account the farming systems, mussels from the EDR site showed the lowest content of C14:0 (*p* < 0.001) compared to mussels from IDR and SIDR farms, evidencing that mussels’ stocking density and, consequently, the environmental conditions may affect the variation in FA content. Effectively, the decrease in SFA content, due to their energetic-type function, indicates a low energetic stress on mollusks [27]. However, it is important to underline that the main SFA detected (C15:0; C16:0; C17:0; C18:0) showed higher values (*p* < 0.001) at the last sampling month in all farming systems analyzed. The greater SFA content found in the mussels collected in June could be related to the high availability of phytoplankton and zooplankton biomass during the warmer months, as previously assumed [20]. Indeed, the phytoplankton contribution and diversity of the marine environments in the mussel diet are the main factors affecting their nutritional composition. Environmental stressors, such as exposure to contaminants, heavy metals, or thermal changes, can also affect the fatty acids profile of bivalves [28,29]. Indeed, when exposed to heat stress, the mussel membrane increases in fluidity resulting in a higher SFA content [30]. Taken all together, the SFA content of the Gargano mussels observed in our study was lower than that reported for the Italian Venice lagoon *Mytilus galloprovincialis* by Bordignon et al. [2]. This result could be related to the Gargano coastal environment, especially to the hydrodynamic conditions that affect water traits and food availability for mussels.

Referring to monounsaturated fatty acids (MUFA), significative differences in the total MUFA content were found in the mussels collected in the Gargano area due to farming system (*p* < 0.01) and sampling time (*p* < 0.001). Mussels from IDR farming site showed a greater palmitoleic acid (C16:1; *p*< 0.001) content in April than those from EDR and SIDR farming sites. It is important to point out that, quantitatively, palmitoleic acid is the main monounsaturated fatty acid in mussels ranging between 9% and 35% of total fatty acids. Considering the sampling time, a lower content of palmitoleic acid was found in mussels collected in June. In line with our findings, the C16:1 has been previously recognized by a greater variability/fluctuation according to the seawater temperature changes [22].

The C18:1t9, C18:1t11, C18:1c9, and C20:1 monounsaturated fatty acids were also affected by the sampling time (*p* < 0.001 for all MUFA considered), showing a greater content in mussels collected at the last sampling month. Our results are in agreement with Vetrella et al. [31] who found an increase in MUFA content mussels in warmer months (spring and summer). In particular, the higher content of MUFA found could be ascribed to the presence and composition of different feed species as well as phytoplankton, zooplankton, and dinoflagellates [32].

Referring to polyunsaturated fatty acids (PUFAs), the sampling time significantly affected the content of all identified PUFAs (*p* < 0.001 for each PUFA) in mussels collected from all farming sites, but with different trends. In particular, a decrease in C18:2t9t12 (linoelaidic acid), C18:3n6 (α-linoleic acid), and C20:5n3 (eicosapentaenoic acid, EPA) was found, due to the sampling time, irrespective of the farming method applied, whereas a significant increase in the content of C18:2c9c12 (linoleic acid), C18:3n3 (α-linolenic), C20:2n6 (eicosadienoic acid), C20:4n6 (arachidonic acid), and C22:6n3 (docosaenoic acid, DHA) was found. These findings could be results of the environmental conditions, particularly the seawater temperature as well as the high level of phytoplankton biomass in the mussels’ diet, as previously assumed [2,24,26,32]. Effectively, mussels ingest and filter large amounts of seawater; therefore, the predominance of beneficial PUFAs in mussels’ tissue found during the warmer seasons could be related to the presence of different feed sources and species (phytoplankton, zooplankton, dinoflagellates, or bacterial particles) characterized by beneficial PUFA composition [2,27].

The conjugated linoleic acids (CLAs) isomers are recognized for their beneficial effects on human health [33]. In our study, a significant increase in the total CLA isomer content was found due to farming systems (*p* < 0.001) and sampling time (*p* < 0.001). Although an increase in the CLA content was found from April to June in mussels collected from all farming systems, mussels from the EDR and SIDR sites exhibited the greatest content of CLA isomers compared to IDR mussels. These results could be imputed to seawater food sources, and especially to the composition of phytoplankton, rich in linoleic (C18:2) and linolenic (C18:3) acids [34], as well as to the environmental factors and greater food availability of the type of farming system characterized by the lowest animal density.

### 3.4. Amino Acid Profile

**Figure 2:**
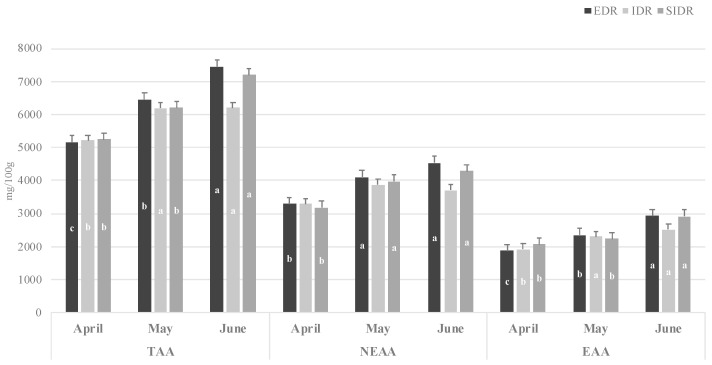
Effect of farming system and sampling time on total amino acid (TAA), non-essential amino acid (NEAA), and essential amino acid (EAA) content (mg/100 g) of *Mytilus galloprovincialis* harvested in the Northern Coastal Area of Gargano. (a, b, c = *p* < 0.05 sampling time effect).

The effect of the farming site and sampling time on the amino acid composition of *Mytilus galloprovincialis* harvested in the Northern Coastal Area of Gargano is reported in Figure 2 and Table 4. A total of nineteen amino acids (total amino acids, TAA) including nine essential amino acids (EAAs: His, Thr, Val, Met, Trp, Phe, Ile, Leu, Lys) and ten non-essential amino acids (NEAAs: Asp, Glu, Ser, Gly, Arg, Ala, Tyr, Cys, Hyp, Pro) were detected in the Gargano mussels. The amino acid profile of mussels (in terms of TAA, NEAA, and EAA concentrations; Figure 2) was significantly affected by the sampling time (*p* < 0.001 in TAA, NEAA, and EAA), showing an increase in their content from April to June, whereas no significant differences were found among the three farming sites analyzed.

On the other hand, when we look at the single/individual amino acid (Table 4), the data evidenced that all the amino acids detected were affected by sampling time with the exception of the tryptophan (Trp) essential amino acid. However, it is important to underline that the sampling time did not affect the content of several amino acids (aspartic acid, glutamic acid, threonine, arginine, alanine, tyrosine, valine, methionine, Isoleucine, and Leucine) in mussels collected from the IDR site. Moreover, an effect of the farming system was found for glutamic acid (Glu; *p* < 0.05), serine (Ser; *p* < 0.05), tryptophan (trp; *p* < 0.001), hydroxyproline (Hyp; *p* < 0.05), and proline (Pro; *p* < 0.01) contents.

Glutamic acid is the primary amino acid in the biochemical metabolism of brain tissue which can help to improve memory, whereas proline and hydroxyproline have a key role in protein structures and the maintenance of cellular redox homeostasis [35,36]. In addition, tryptophan, which is an EAA, is a serotonin/neurotransmitter precursor involved in the stress response. The amino acids profile of the Gargano mussels from EDR and SIDR sites evidences that the farming system contributes to ameliorating the nutritional value of mussels’ tissue.

Taking into account the sampling time, an increase in the content of all the amino acids detected was found (*p* < 0.001) with the highest values in mussels collected at the last sampling month (June). These findings are to some extent in line with previous knowledge [15] where a remarkable fluctuation in amino acid content in Mediterranean mussels cultivated in the Gulf of Trieste (North Italy) was observed due to environmental and nutritional nitrogen-related composition of seawater. The increase in amino acids content of mussels during the spring season confirms that the climatic conditions and the variability in the seawater temperature in the Mediterranean Gargano area can have a positive impact on the nutritional composition of mussels.

## 4. Conclusions

In summary, the results of the present study showed that Mediterranean mussels cultivated in the Northern Coastal Area of Gargano are products with an excellent nutritional profile characterized by low fat and high PUFA and amino acid contents. The results from this study suggest that mussels’ stocking density contributes to increasing the content of compounds with high nutritional value in the Mediterranean Gargano environmental and ecosystem conditions from April to June.

Differences were detected in mussels collected in the Gargano area, evidencing a better nutritional profile, in terms of higher content of CLA isomers and glutamic acid, serine, tryptophan, hydroxyproline, and proline amino acids, of mussels cultivated in extensive and semi-intensive farming conditions compared to mussels collected from intensive rearing systems. Further supporting evidence is warranted to define seasonal variations in mussels’ nutritional composition and to identify appropriate farming strategies and practices in the Gargano area to improve mussels’ sustainable production under climate changes.

## Figures and Tables

**Table 1:** Environmental seawater parameters (mean ± SE) of the three farming sites recorded in three months during the spring season.

		Farming System ^a^		
	Sampling Time	EDR	IDR	SIDR
	April	8.16 ± 0.02	8.23 ± 0.02	8.20 ± 0.02
pH	May	8.28 ± 0.01	8.23 ± 0.02	8.44 ± 0.02
	June	8.63 ± 0.02	8.62 ± 0.01	8.62 ± 0.02
	April	15.27 ± 0.04	15.12 ± 0.06	15.15 ± 0.04
Temperature (°C)	May	17.21 ± 0.06	17.36 ± 0.04	17.30 ± 0.05
	June	24.90 ± 0.02	24.70 ± 0.08	24.70 ± 0.04
	April	30.50 ± 0.01	30.80 ± 0.01	30.30 ± 0.02
Salinity (PSU_g/kg_ppt)	May	29.70 ± 0.02	30.10 ± 0.01	29.00 ± 0.01
	June	25.99 ± 0.04	27.76 ± 0.02	25.73 ± 0.03
	April	109.99 ± 0.03	109.79 ± 0.04	112.51 ± 0.04
DO ^b^ (% Sat)	May	114.40 ± 0.04	114.19 ± 0.04	117.03 ± 0.06
	June	125.0 ± 0.05	120.5 ± 0.06	121.1 ± 0.08

^a^ EDR: 70 m distance between backbones, 90 cm distance between dropper ropes; IDR: 30 m distance between backbones, 60/70 cm distance between dropper rope; and SIDR: 70 m distance between backbones, 50/60 cm distance between dropper rope. ^b^ DO: dissolved oxygen.

**Table 2:** Effect of farming system and sampling time on chemical composition (%) of *Mytilus galloprovincialis* harvested in the Northern Coastal Area of Gargano (means ± SEM).

		Farming System ^a^							Effects, *p*		
	Sampling Time	EDR		IDR		SIDR		SEM	Farming System	Sampling Time	Farming System × Sampling Time
	April	2.100	Ba	2.622	Aa	2.401	ABa	0.15	**	***	NS
Fat	May	2.232	a	2.487	a	2.120	a				
	June	1.244	Bb	1.846	Ab	1.482	ABb				
	April	8.303	b	8.378	b	8.135	b	0.29	NS	***	NS
Protein	May	10.535	a	10.135	a	9.913	a				
	June	10.445	a	10.775	a	10.620	a				
	April	3.411	a	3.231	a	2.869	a	0.27	NS	***	NS
Ash	May	2.287	ab	2.858	ab	2.210	ab				
	June	1.920	b	2.244	b	2.011	b				
	April	79.643	Ba	79.310	Ba	81.448	Aa	0.35	***	***	*
Moisture	May	77.554	b	76.620	b	77.140	b				
	June	78.190	Ab	76.590	Bb	77.705	Ab				
	April	6.543	b	6.460	b	5.148	b	0.44	NS	***	NS
Carbohydrate	May	7.392	ab	7.901	a	8.618	a				
	June	8.201	a	8.545	a	8.183	a				

^a^ EDR: 70 m distance between backbones, 90 cm distance between dropper ropes; IDR: 30 m distance between backbones, 60/70 cm distance between dropper rope; and SIDR: 70 m distance between backbones, 50/60 cm distance between dropper rope. NS = not significant; * *p* < 0.05; ** *p* < 0.01; *** *p* < 0.001. a, b = *p* < 0.05 in the column (sampling time effect); A, B = *p* < 0.05 in the row (farming system effect).

**Table 3:** Effect of farming system and sampling time on fatty acid profile (g/100 g, %) of *Mytilus galloprovincialis* harvested in the Northern Coastal Area of Gargano (means ± SEM).

		Farming System ^a^						SEM	Effects, *p*		
	Sampling Time	EDR		IDR		SIDR			Farming System	Sampling Time	Farming System × Sampling Time
	April	7.46	Ba	8.20	Aa	7.11	Ba	0.20	***	***	***
C14:0	May	6.24	Bb	6.57	Bb	7.56	Aa				
	June	4.19	Bc	5.55	Ac	4.98	Ab				
	April	0.33	c	0.34	b	0.35	c	0.01	NS	***	NS
C15:0	May	0.38	b	0.36	b	0.39	b				
	June	0.49	a	0.48	a	0.49	a				
	April	9.91	c	10.48	c	10.03	b	0.21	**	***	NS
C16:0	May	11.60	Ab	11.31	Ab	10.47	Bb				
	June	13.74	a	13.98	a	13.38	a				
	April	3.03	Ac	2.12	Bc	3.06	Ab	0.11	***	***	***
C17:0	May	3.37	Ab	3.01	Bb	2.48	Cc				
	June	4.82	Aa	4.22	Ba	4.61	Aa				
	April	1.33	c	1.13	c	1.26	c	0.04	***	***	*
C18:0	May	1.65	Ab	1.54	Ab	1.42	Bb				
	June	2.35	a	2.28	a	2.27	a				
	April	1.27	Aa	1.09	Ba	1.37	Aa	0.05	***	***	***
Other SFA	May	0.98	Bb	0.94	Bb	1.16	Ab				
	June	0.70	c	0.73	c	0.67	c				
	April	14.63	Ba	18.22	Aa	14.84	Ba	0.44	***	***	**
C16:1	May	13.75	a	14.81	b	14.80	a				
	June	9.69	b	10.40	c	10.46	b				
	April	0.19	b	0.32	a	0.31	b	0.05	NS	***	NS
C18:1t9	May	0.17	b	0.15	b	0.17	b				
	June	0.46	a	0.31	a	0.48	a				
	April	0.89	b	0.93	b	0.84	b	0.05	NS	***	*
C18:1t11	May	0.89	Bb	0.93	Bb	1.13	Aa				
	June	1.26	Ba	1.44	Aa	1.24	Ba				
	April	1.91	c	2.01	b	2.02	c	0.09	NS	***	NS
C18:1c9	May	2.26	b	2.21	b	2.37	b				
	June	2.60	a	2.74	a	2.73	a				
	April	0.78	Ab	0.59	Bc	0.79	Ab	0.04	**	***	NS
C20:1	May	0.84	b	0.73	b	0.81	b				
	June	1.28	a	1.19	a	1.13	a				
Other MUFA	April	1.20	Ba	1.31	Aa	1.18	Ba	0.03	**	***	*
	May	0.91	Bb	0.98	ABb	1.04	Ab				
	June	0.69	c	0.74	c	0.75	c				
	April	0.53	a	0.58	a	0.62	a	0.03	NS	***	NS
C18:2t9t12	May	0.27	b	0.29	b	0.34	b				
	June	0.07	c	0.06	c	0.06	c				
	April	0.70	b	0.69	b	0.65	b	0.08	NS	***	NS
C18:2c9c12	May	0.83	b	0.69	b	0.85	b				
	June	1.39	a	1.43	a	1.35	a				
	April	0.15	Ba	0.16	Ba	0.23	Aa	0.01	***	***	***
C18:3n6	May	0.13	Ba	0.12	Bb	0.17	Ab				
	June	0.09	Bb	0.14	Aab	0.10	Bc				
	April	0.92	b	0.95	b	0.87	b	0.07	NS	***	NS
C18:3n3	May	0.98	b	0.99	b	1.04	b				
	June	1.68	a	1.80	a	1.68	a				
	April	0.19	Bb	0.15	Bb	0.34	A	0.03	*	***	**
C20:2n6	May	0.24	b	0.21	b	0.27					
	June	0.38	a	0.40	a	0.34					
	April	1.51	Ab	1.20	Bc	1.33	ABc	0.08	**	***	NS
C20:4n6	May	1.69	b	1.51	b	1.57	b				
	June	2.73	ABa	2.54	Ba	2.90	Aa				
	April	38.43	a	37.57	a	39.54	a	1.21	NS	***	NS
C20:5n3	May	36.10	a	37.35	a	36.86	a				
	June	25.80	b	24.19	b	25.86	b				
	April	12.71	b	10.43	c	11.07	b	0.87	NS	***	NS
C22:6n3	May	14.48	b	13.29	b	12.95	b				
	June	23.06	a	23.13	a	22.03	a				
	April	0.15	a	0.10		0.26	a	0.01	NS	***	**
Other PUFA	May	0.07	b	0.12		0.14	b				
	June	0.10	ab	0.09		0.06	c				
	April	23.33	b	23.34	b	23.17	b				
Total SFA	May	24.23	b	23.74	b	23.48	b	0.35	NS	***	NS
	June	26.29	a	27.23	a	26.39	a				
	April	19.59	Ba	23.38	Aa	19.98	Ba				
Total MUFA	May	18.82	a	19.82	b	20.33	a	0.53	***	***	**
	June	15.98	b	16.82	c	16.81	b				
	April	57.08	A	53.28	Bb	56.84	A				
Total PUFA	May	56.96		56.44	a	56.19		0.55	***	*	*
	June	57.73	A	55.95	Ba	56.80	AB				
	April	1.77	Ac	1.44	Bc	1.94	Ab	0.07	***	***	NS
CLA	May	2.16	Ab	1.87	Bb	2.00	ABb				
	June	2.44	Aa	2.16	Ba	2.42	Aa				

^a^ EDR: 70 m distance between backbones, 90 cm distance between dropper ropes; IDR: 30 m distance between backbones, 60/70 cm distance between dropper rope; and SIDR: 70 m distance between backbones, 50/60 cm distance between dropper rope. NS = not significant; * *p* < 0.05; ** *p* < 0.01; *** *p* < 0.001. a, b, c = *p* < 0.05 in the column (sampling time effect); A, B = *p* < 0.05 in the row (farming system effect).

**Table 4:** Effect of farming system and sampling time on amino acid content (mg/100g) of Gargano of *Mytilus galloprovincialis* harvested in the Northern Coastal Area of Gargano (means ± SEM).

	Sampling Time	Farming System ^a^						SEM	Effects, *p*		
		EDR		IDR		SIDR			Farming System	Sampling Time	Farming System × Sampling Time
Aspartic acid (Asp)	April	539.5	b	515.28		476.31	b	45.17	NS	***	NS
	May	662.36	ab	623.05		691.56	a				
	June	677.19	a	613.53		657.62	a				
Glutamic acid (Glu)	April	769.35	Ab	751.80	A	614.92	Bb	40.73	**	***	NS
	May	945.64	Aa	822.91	B	890.81	Ba				
	June	955.89	Aa	799.12	B	837.36	ABa				
Serine (Ser)	April	210.82	b	219.06	b	243.93	b	18.02	*	***	NS
	May	272.1	ab	282.85	a	301.23	a				
	June	318.81	Aa	245.47	Bab	317.7	Aa				
Histidine (His)	April	133.52	c	171.69	c	130.92	c	21.69	NS	***	NS
	May	296.17	b	258.85	b	245.92	b				
	June	429.59	a	458.86	a	518.06	a				
Glycine (Gly)	April	333.37	b	344.35	b	379.41	b	30.25	NS	***	NS
	May	404.92	b	446.64	a	393.11	ab				
	June	538.73	a	360.6	ab	474.18	a				
Threonine (Thr)	April	221.15	b	230.25		264.04		22.02	NS	***	NS
	May	280.61	b	293.91		286.87					
	June	368.82	a	286.36		325.49					
Arginine (Arg)	April	506.05	b	499.58		496.93	b	42.06	NS	***	NS
	May	546.74	b	540.8		602.89	b				
	June	699.38	a	553.87		759.51	a				
Alanine (Ala)	April	362.79	b	380.26		382.35		24.34	NS	*	NS
	May	454.62	a	422.59		400.26					
	June	417.01	ab	354.43		401.59					
Tyrosine (Tyr)	April	210.37	b	215.7		191.4	b	20.49	NS	***	NS
	May	294.53	a	266.62		251.03	ab				
	June	291.49	a	237.95		283.32	a				
Cysteine (Cys)	April	159.79	b	158.94	b	186.8	b	17.17	NS	***	NS
	May	215.89	a	218.44	a	213.58	ab				
	June	245.01	a	216.78	a	251.24	a				
Valine (Val)	April	184.98	b	201.9		180.67	b	15.82	NS	***	NS
	May	219.02	b	218.94		218.12	ab				
	June	274.14	a	224.17		258.99	a				
Methionine (Met)	April	168.33	b	168.55		192.22		16.09	NS	**	NS
	May	205.66	ab	212.49		226.59					
	June	248.43	a	194.66		218.3					
Tryptophan (Trp)	April	114.74	B	104.33	B	192.75	Aa	10.99	***	NS	NS
	May	127.71		101.9		130.75	b				
	June	128.29	AB	100.82	B	135.61	Ab				
Phenylalanine (Phe)	April	185.71	b	182.32	b	221.87	b	21.56	NS	***	NS
	May	225.69	b	219.69	ab	226.29	b				
	June	293.22	a	248.01	a	291.99	a				
Isoleucine (Ile)	April	228.45	b	225.39		249.78	ab	15.81	NS	**	NS
	May	247.73	b	242.28		222.44	b				
	June	297.65	a	254.58		295.99	a				
Leucine (Leu)	April	284.46	b	285.04		289.49		18.09	NS	***	NS
	May	334.07	ab	336.45		285.49					
	June	387.91	a	316		389.74					
Lysine (Lys)	April	346.48	c	348.28	b	348.73	b	19.63	NS	***	NS
	May	417.09	b	418.02	a	392.4	b				
	June	493.39	a	423.67	a	479.99	a				
Hydroxiproline (Hyp)	April	10.60	c	12.41	b	9.70	b	1.06	*	***	*
	May	14.16	ABb	15.85	Aa	12.06	Bb				
	June	23.11	Aa	17.81	Ba	19.24	Ba				
Proline (Pro)	April	186.64	c	192.01	b	188.45	b	21.83	*	***	NS
	May	297.66	Ab	239.74	ABb	212.06	Bb				
	June	363.74	Aa	305.8	ABa	282.08	Ba				

^a^ EDR: 70 m distance between backbones, 90 cm distance between dropper ropes; IDR: 30 m distance between backbones, 60/70 cm distance between dropper rope; and SIDR: 70 m distance between backbones, 50/60 cm distance between dropper rope. NS = not significant; * *p* < 0.05; ** *p* < 0.01; *** *p* < 0.001. a, b, c = *p* < 0.05 in the column (sampling time effect); A, B = *p* < 0.05 in the row (farming system effect).

## Data Availability

The original contributions presented in the study are included in the article, further inquiries can be directed to the corresponding author.
